# Dressed state dynamics of two-component Bose-Einstein Condensates in state-dependent potentials

**DOI:** 10.1038/s41598-018-22582-5

**Published:** 2018-03-14

**Authors:** Qinzhou Ye, Jiahao Huang, Min Zhuang, Honghua Zhong, Chaohong Lee

**Affiliations:** 1 Laboratory of Quantum Engineering and Quantum Metrology, School of Physics and Astronomy, Sun Yat-Sen University (Zhuhai Campus), Zhuhai, 519082 China; 2 Key Laboratory of Optoelectronic Materials and Technologies, Sun Yat-Sen University, (Guangzhou Campus), Guangzhou, 510275 China

## Abstract

Dressed potentials realized by coupling state-dependent bare potentials with external fields have important applications in trapping and manipulating atoms. Here, we study the dynamics of dressed states for coupled two-component Bose-Einstein condensates (BECs) in state-dependent potentials. Through both analytical and numerical methods, we find that the dressed state dynamics sensitively depend on both the inter-component coupling strength and the initial state. If the inter-component coupling is strong enough and the initial wave packet is located at the potential minimum, the dressed states can be decoupled and the Josephson oscillations and macroscopic quantum self-trapping appear. However, if the initial wave packet is located far away from the potential minimum, the wave packet will acquire a large kinetic energy and Landau-Zener transitiozs between the dressed states occur at the avoided-crossing point. Further, we give the validity ranges and conditions for the formation of adiabatic potentials, where the influences of Landau-Zener transitions can be ignored. Our results give an insight on how the inter-component coupling affects the dressed state dynamics and how to realize adiabatic potentials with BECs in state-dependent potentials.

## Introduction

Laser, radio-frequency (rf) and static external fields are promising tools for trapping and manipulating cold atoms. The control of ultracold atomic systems provides great opportunities for developing quantum technology such as quantum metrology^1–4^, quantum engineering^5,6^ and quantum simulation^7^. Among these investigations, field-induced adiabatic dressed potentials created by a versatile combination of rf and static magnetic fields played an important role^8,9^. Dressed potentials arise when two or more internal spin states that experience different potentials are coupled by an external field. The motions of the trapped atoms are no longer determined by the individual bare potentials but become dominated by the dressed potentials dependent on the bare potentials and the inter-component couplings. If there is no any Landau-Zener (LZ) transition induced by the kinetic energy of the atoms, the dressed potentials are referred as adiabatic potentials. Adiabatic potentials can avoid the limitation of magnetic traps and can be used for realizing traps with different geometries, including double-well traps^10^, ring traps^11,12^, lattice potentials^13,14^ and so on. Applications of adiabatic potentials include exploring novel properties of superfluidity^15,16^ and serving as beam splitters in matter-wave interferometry^10^. In atom chips^17,18^, adiabatic potentials can be tailored more freely for trapping and manipulating atoms on micrometer scale, which can be useful for quantum sensing^19,20^.

Recently, many theoretical and experimental works focus on how to realize different geometries of adiabatic potentials^21^. To tailor a desired adiabatic potential, the strength of the induced rf field (i.e., the inter-component coupling) needs to be carefully controlled according to the geometry of bare potentials^9^. One of the important tools in atom chips is the combined coherent manipulation of the internal and motional states via state-dependent potentials. By dressing different atomic spin states with a spatially varying field, state-dependent potentials have been realized with atomic BECs^22^. The state-dependent potentials enable one to control the inter-state interactions by tuning the overlap of two internal states, which are a crucial ingredient for entanglement generation^23^, atom-chip quantum gate^24,25^ and spin squeezing^26^. For atomic BECs in state-dependent potentials, the specific adiabatic potential in the double-well shape is an important element for quantum engineering. If the strength of the rf coupling is too strong, the double-well structure would be vanished in the dressed potential. However, if the strength of the rf coupling is too weak, the gap between the dressed potentials would be small and non-adiabatic transition between the dressed potentials becomes significant. It is essential to know how the interplay between inter-component coupling and state-dependent potentials influences the formation of adiabatic potentials. Therefore, two natural questions arise: (i) what is the validity ranges for realizing adiabatic potentials with atomic BECs in state-dependent potentials? and (ii) how the dressed state dynamics behave under different inter-component coupling?

In this article, we consider two-component BECs in state-dependent harmonic potentials, which are coupled by an external field. The separation of the minima of the two different harmonic traps can be tuned by using additional spatially varying microwave or magnetic field in experiments^22,27^. By applying a coupling field, the two independent single-well potentials become an upper single-well potential and a lower double-well potential in the dressed state picture. We will demonstrate that, the dressed state dynamics sensitively depend on the induced coupling strength and the selection of the initial state.

When the coupling strength is sufficiently strong, we observe Josephson oscillations of the dressed states. As the coupling strength becomes weaker, Josephson oscillations of the dressed states gradually change. We show that, the periods and the critical condition of macroscopic quantum self-trapping (MQST) are dramatically influenced by the coupling strength. In addition, even for a strong coupling strength, if the initial wave packet located at a position far away from the minimum, the dressed state will acquire a large kinetic energy, which leads to the occurrence of LZ transitions. We find that the transition rate will become smaller as the initial location getting closer to the potential minimum, which can be well explained by the LZ formula. Under certain coupling strengths, we give the corresponding validity ranges and conditions for the formation of adiabatic potential where the influences of LZ transitions can be avoided.

## Results

### Model

The system we study is coupled two-component BECs trapped in state-dependent potentials. The state-dependent potentials can be feasibly induced by a spatially varying microwave^22^ or a magnetic field gradient^27^. The condensed atoms in two different hyperfine levels experience different harmonic potentials with a spatial separation between the two potential minima. The two hyperfine states are split by an applied magnetic field, and are coupled by a driving field with detuning δ = ω − ω_0_ (ω_0_ is the transition frequency between the two hyperfine states and ω is the oscillation frequency of the induced field) and coupling strength Ω. Under the rotating wave approximation and treating the atom-atom interaction within the mean-field (MF) theory, the system can be well described by the coupled Gross-Pitaevskii equations (CGPEs):

1 $$\begin{array}{rcl}{\rm{i}}\hslash \frac{\partial }{\partial {\rm{t}}}{{\rm{\Psi }}}_{1}({\bf{r}},{\rm{t}}) & = & [-\frac{{\hslash }^{2}}{2{\rm{m}}}{\nabla }^{2}+{{\rm{V}}}_{1}({\bf{r}})+{{\rm{\beta }}}_{11}{|{{\rm{\Psi }}}_{1}({\bf{r}},{\rm{t}})|}^{2}+{{\rm{\beta }}}_{12}{|{{\rm{\Psi }}}_{2}({\bf{r}},{\rm{t}})|}^{2}+\frac{{\rm{\delta }}}{2}]{{\rm{\Psi }}}_{1}({\bf{r}},{\rm{t}})+{{\rm{\Omega }}{\rm{\Psi }}}_{2}({\bf{r}},{\rm{t}}),\\ {\rm{i}}\hslash \frac{\partial }{\partial {\rm{t}}}{{\rm{\Psi }}}_{2}({\bf{r}},{\rm{t}}) & = & [-\frac{{\hslash }^{2}}{2{\rm{m}}}{\nabla }^{2}+{{\rm{V}}}_{2}({\bf{r}})+{{\rm{\beta }}}_{12}{|{{\rm{\Psi }}}_{1}({\bf{r}},{\rm{t}})|}^{2}+{{\rm{\beta }}}_{22}{|{{\rm{\Psi }}}_{2}({\bf{r}},{\rm{t}})|}^{2}-\frac{{\rm{\delta }}}{2}]{{\rm{\Psi }}}_{2}({\bf{r}},{\rm{t}})+{{\rm{\Omega }}{\rm{\Psi }}}_{1}({\bf{r}},{\rm{t}}).\end{array}$$

Here, Ψ_1,2_(**r**, t) are the condensate wave functions of the two components and m is the mass of the atom. ${{\rm{V}}}_{1}({\bf{r}})=\frac{1}{2}{\rm{m}}[{{\rm{\omega }}}_{{\rm{x}}}^{2}{({\rm{x}}-{{\rm{x}}}_{0})}^{2}+{{\rm{\omega }}}_{{\rm{y}}}^{2}{{\rm{y}}}^{2}+{{\rm{\omega }}}_{{\rm{z}}}^{2}{{\rm{z}}}^{2}]$ and ${{\rm{V}}}_{2}({\bf{r}})=\frac{1}{2}{\rm{m}}[{{\rm{\omega }}}_{{\rm{x}}}^{2}{({\rm{x}}+{{\rm{x}}}_{0})}^{2}+{{\rm{\omega }}}_{{\rm{y}}}^{2}{{\rm{y}}}^{2}+{{\rm{\omega }}}_{{\rm{z}}}^{2}{{\rm{z}}}^{2}]$ are the state-dependent potentials with 2x_0_ being the spatial separation between the two potential minima. ${{\rm{\beta }}}_{{\rm{ij}}}=4{\rm{\pi }}{\hslash }^{2}{{\rm{a}}}_{{\rm{ij}}}/m\,({\rm{i}},{\rm{j}}=1,2)$ is the interaction constant with a_ij_ being the s-wave scattering length between i-th and j-th components.

We consider an elongated system of condensed atoms. That is, the atoms are confined by a strong transverse confinement ω_y_, ω_z_ ≫ ω_x_. Integrating the transverse coordinates, the elongated system can be described by the one-dimensional (1D) CGPEs:

2 $$\begin{array}{rcl}{\rm{i}}\frac{\partial }{\partial {\rm{t}}}{{\rm{\Psi }}}_{1}({\rm{x}},{\rm{t}}) & = & [-\frac{1}{2}\frac{{\partial }^{2}}{\partial {{\rm{x}}}^{2}}+{{\rm{V}}}_{1}({\rm{x}})+{{\rm{g}}}_{11}{|{{\rm{\Psi }}}_{1}({\rm{x}},{\rm{t}})|}^{2}+{{\rm{g}}}_{12}{|{{\rm{\Psi }}}_{2}({\rm{x}},{\rm{t}})|}^{2}+\frac{{\rm{\delta }}}{2}]{{\rm{\Psi }}}_{1}({\rm{x}},{\rm{t}})+{{\rm{\Omega }}{\rm{\Psi }}}_{2}({\rm{x}},{\rm{t}}),\\ {\rm{i}}\frac{\partial }{\partial {\rm{t}}}{{\rm{\Psi }}}_{2}({\rm{x}},{\rm{t}}) & = & [-\frac{1}{2}\frac{{\partial }^{2}}{\partial {{\rm{x}}}^{2}}+{{\rm{V}}}_{2}({\rm{x}})+{{\rm{g}}}_{12}{|{{\rm{\Psi }}}_{1}({\rm{x}},{\rm{t}})|}^{2}+{{\rm{g}}}_{22}{|{{\rm{\Psi }}}_{2}({\rm{x}},{\rm{t}})|}^{2}-\frac{{\rm{\delta }}}{2}]{{\rm{\Psi }}}_{2}({\rm{x}},{\rm{t}})+{{\rm{\Omega }}{\rm{\Psi }}}_{1}({\rm{x}},{\rm{t}}),\end{array}$$

in which ${{\rm{V}}}_{1}=\frac{1}{2}{({\rm{x}}-{{\rm{x}}}_{0})}^{2},{{\rm{V}}}_{2}=\frac{1}{2}{({\rm{x}}+{{\rm{x}}}_{0})}^{2}$ and ${{\rm{g}}}_{{\rm{ij}}}=2{{\rm{a}}}_{{\rm{ij}}}{({{\rm{\omega }}}_{{\rm{y}}}{{\rm{\omega }}}_{{\rm{z}}})}^{1/2}{{\rm{\omega }}}_{{\rm{x}}}^{-1}{{\rm{a}}}_{{\rm{H}}}^{-1}\,({\rm{i}},{\rm{j}}=1,2)$. The condensate wave functions are normalized to give the populations ${{\rm{N}}}_{{\rm{i}}}({\rm{t}})=\int {\rm{dx}}{|{{\rm{\Psi }}}_{{\rm{i}}}({\rm{x}},{\rm{t}})|}^{2}({\rm{i}}=1,2)$, where the total number N = N_1_ + N_2_ is constant. Here, time is in units of ${{\rm{t}}}_{{\rm{s}}}={{\rm{\omega }}}_{{\rm{x}}}^{-1}$, the spatial distance is in units of ${{\rm{x}}}_{{\rm{s}}}={(\hslash /{m{\rm{\omega }}}_{{\rm{x}}})}^{1/2}$, the wave function amplitude is in units of ${{\rm{x}}}_{{\rm{s}}}^{-3/2}\,$and the energy is in units of ${{\rm{E}}}_{{\rm{s}}}=\hslash {{\rm{\omega }}}_{{\rm{x}}}$. To relate the dimensionless parameters with some typical experiments, we take ^87^Rb atoms for an example. The mass of the atom m = 1.44 × 10^−25^ kg, the scattering lengths a_11_ = a_12_ = a_22_ = 5.3 nm, and the trapping frequencies ω_x_ = 2π × 109 Hz, ω_y_ = ω_z_ = 2π × 500 Hz. For hyperfine states |${\rm{F}}=1,{{\rm{m}}}_{{\rm{F}}}=-1\rangle$ and |${\rm{F}}=2,{{\rm{m}}}_{{\rm{F}}}=1\rangle$, the state-dependent potentials split along x with the distance in the range of 0–9.4 μm^22^ and the Raman coupling strength varies between zero and 2π × 625 Hz in experiments^28^. Setting $\hslash ={\rm{m}}={{\rm{\omega }}}_{{\rm{x}}}=1$, one can obtain the corresponding ranges of the dimensionless parameters: 0 ≤ Ω ≤ 5.73, 0 ≤ x_0_ ≤ 4.56 and g_11_ = g_22_ = g_12_ = g = 0.0471. In our calculation, we assume that the intra-component and inter-component interaction strengths are equal. Since the interaction strengths can be tuned by Feshbach resonances in experiments^29^, we will vary g to study the nonlinear effects. In addition, we only consider the on-resonance case, where δ = 0.

### Dressed potentials

Transforming the bare states to a new basis that diagonalizes the bare potentials V_1_ and V_2_ and the coupling Ω at each spatial point x, one can obtain a set of spatially dependent eigenstates which are the dressed states. Correspondingly, the spatially varying eigen-energies constitute the so-called dressed potentials. The diagonalization can be done by a unitary matrix U, where

3 $${{\rm{U}}}^{\dagger }[\begin{array}{cc}{{\rm{V}}}_{1} & {\rm{\Omega }}\\ {\rm{\Omega }} & {{\rm{V}}}_{2}\end{array}]{\rm{U}}=[\begin{array}{cc}{{\rm{V}}}_{+} & 0\\ 0 & {{\rm{V}}}_{-}\end{array}].$$

After the diagonalization, the dressed potentials can be obtained as

4 $${{\rm{V}}}_{\pm }=\frac{{{\rm{x}}}^{2}+{{\rm{x}}}_{0}^{2}}{2}\pm \sqrt{{{\rm{x}}}_{0}^{2}{{\rm{x}}}^{2}+{{\rm{\Omega }}}^{2}}.$$

**Figure 1:**
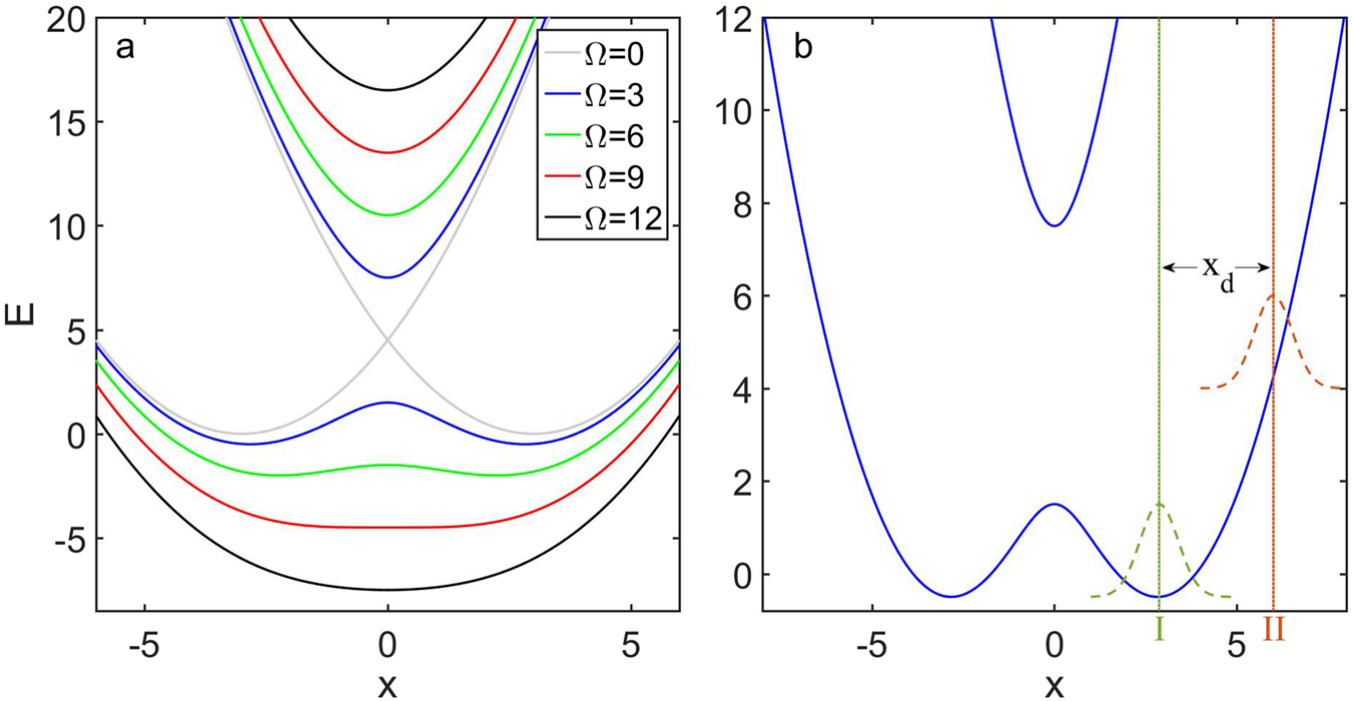
Dressed potentials and initial states. (**a**) Dressed potentials for different coupling strength Ω with fixed separation of two bare potential minima x_0_ = 3. Here, ${\rm{\Omega }}={{\rm{x}}}_{0}^{2}$ is the critical value for the existence of double well structure in lower dressed potential. (**b**) The blue solid lines indicate the dressed potentials for x_0_ = 3, Ω = 3. The dashed wave packets illustrate the different locations of initial states we adopt in studying BJ dynamics (I. green) and LZ dynamics (II. red). x_d_ is the distance between the centering of the initial wave packet and the minimum of the right well of the lower dressed potential.

For a fixed x_0_, the shape of the above dressed potentials for different Ω are shown in Fig. 1(a). It is obvious that, the gap between the upper and lower dressed potentials becomes larger when the coupling strength Ω increases. When Ω is small, the lower dressed potential is in the shape of double-well while the upper dressed potential remains a single-well structure. However, as Ω grows larger, the barrier height of the lower double-well decreases. When the coupling strength Ω exceeds the critical value ${{\rm{x}}}_{0}^{2}$, the lower dressed potential will change from a double-well structure to a single-well structure. It is clear that, to form an adiabatic double-well potential, there is a tradeoff between the gap of the two dressed potentials and the barrier height of the double-well when x_0_ is fixed.

After the transformation (3), the CGPEs (2) become

5 $$\begin{array}{rcl}{\rm{i}}\frac{\partial }{\partial {\rm{t}}}{{\rm{\Psi }}}_{+} & = & [-\frac{1}{2}\frac{{\partial }^{2}}{\partial {{\rm{x}}}^{2}}+{{\rm{V}}}_{+}+{{\rm{V}}}_{{\rm{kin}}}+{\rm{g}}{|{{\rm{\Psi }}}_{+}|}^{2}+{\rm{g}}{|{{\rm{\Psi }}}_{-}|}^{2}]{{\rm{\Psi }}}_{+}+{{\rm{V}}}_{{\rm{c}}}{{\rm{\Psi }}}_{-},\\ {\rm{i}}\frac{\partial }{\partial {\rm{t}}}{{\rm{\Psi }}}_{-} & = & [-\frac{1}{2}\frac{{\partial }^{2}}{\partial {{\rm{x}}}^{2}}+{{\rm{V}}}_{-}+{{\rm{V}}}_{{\rm{kin}}}+{\rm{g}}{|{{\rm{\Psi }}}_{+}|}^{2}+{\rm{g}}{|{{\rm{\Psi }}}_{-}|}^{2}]{{\rm{\Psi }}}_{-}-{{\rm{V}}}_{{\rm{c}}}{{\rm{\Psi }}}_{+}.\end{array}$$

with

6 $$\begin{array}{rcl}{{\rm{V}}}_{{\rm{kin}}}({\rm{x}}) & = & \frac{{{\rm{x}}}_{0}^{2}{{\rm{\Omega }}}^{2}}{8{({{\rm{x}}}_{0}^{2}{{\rm{x}}}^{2}+{{\rm{\Omega }}}^{2})}^{2}},\\ {{\rm{V}}}_{{\rm{c}}}({\rm{x}},{\partial }_{{\rm{x}}}) & = & {{\rm{C}}}_{1}+{{\rm{C}}}_{2}{\partial }_{{\rm{x}}},\\ {{\rm{C}}}_{1} & = & -\frac{{{\rm{\Omega }}{\rm{x}}}_{0}^{3}{\rm{x}}}{2{({{\rm{x}}}_{0}^{2}{{\rm{x}}}^{2}+{{\rm{\Omega }}}^{2})}^{2}},\quad {{\rm{C}}}_{2}=\frac{{{\rm{\Omega }}{\rm{x}}}_{0}}{2({{\rm{x}}}_{0}^{2}{{\rm{x}}}^{2}+{{\rm{\Omega }}}^{2})}\,.\end{array}$$

where ∂_x_ is the partial derivative with respect to x and Ψ_±_ are the condensate wave functions of the two dressed states. In Eq. (5), one can see that the two dressed states are coupled via the term V_c_ which comes from the kinetic term $-\frac{1}{2}\frac{{\partial }^{2}}{\partial {{\rm{x}}}^{2}}$ in the bare state basis. The coupling between the dressed states is now determined by the coefficients C_1_, C_2_ and ∂_x_Ψ_−_ (∂_x_Ψ_+_). According to Eq. (6), the absolute values of coefficients C_1_ and C_2_ decrease as Ω increases near the avoided-crossing.

The terms ${\partial }_{{\rm{x}}}{{\rm{\Psi }}}_{-}$ and ${\partial }_{{\rm{x}}}{{\rm{\Psi }}}_{+}$ are proportional to the velocity of the dressed states. Provided that the coupling strength Ω is strong enough and the velocity of the states is small enough, the coupling term V_c_ becomes sufficiently small to be neglected. In this case, atoms can be trapped in lower (upper) dressed potential and they can hardly be transferred to the upper (lower) dressed states via LZ transitions. Then the dressed potentials can be regarded as the so-called adiabatic potentials and the system can be described by the following decoupled equations:

7 $$\begin{array}{rcl}{\rm{i}}\frac{\partial }{\partial {\rm{t}}}{{\rm{\Psi }}}_{+} & = & [-\frac{1}{2}\frac{{\partial }^{2}}{\partial {{\rm{x}}}^{2}}+{{\rm{V}}}_{+}+{{\rm{V}}}_{{\rm{kin}}}+{\rm{g}}{|{{\rm{\Psi }}}_{+}|}^{2}+{\rm{g}}{|{{\rm{\Psi }}}_{-}|}^{2}]{{\rm{\Psi }}}_{+},\\ {\rm{i}}\frac{\partial }{\partial {\rm{t}}}{{\rm{\Psi }}}_{-} & = & [-\frac{1}{2}\frac{{\partial }^{2}}{\partial {{\rm{x}}}^{2}}+{{\rm{V}}}_{-}+{{\rm{V}}}_{{\rm{kin}}}+{\rm{g}}{|{{\rm{\Psi }}}_{+}|}^{2}+{\rm{g}}{|{{\rm{\Psi }}}_{-}|}^{2}]{{\rm{\Psi }}}_{-},\end{array}$$

where the dynamics of the atoms in one of the dressed states are solely dependent on the corresponding dressed potential. However, if the coupling between the two dressed states V_c_ is not neglectable, the dynamics of the upper (lower) dressed states will be affected by the lower (upper) dressed state, and one can only use the CGPEs (5) to study the system’s dynamics.

In the following, we treat the system as multiple coupled modes of macroscopic matter waves. If the barrier of the lower dressed potential is sufficiently high, we can expand the condensate wave function of the lower dressed state and the upper dressed state as:

8 $$\begin{array}{rcl}{{\rm{\Psi }}}_{-}({\rm{x}},{\rm{t}}) & = & {{\rm{\psi }}}_{1}({\rm{t}}){{\rm{\varphi }}}_{1}({\rm{x}})+{{\rm{\psi }}}_{2}({\rm{t}}){{\rm{\varphi }}}_{2}({\rm{x}}),\\ {{\rm{\Psi }}}_{+}({\rm{x}},{\rm{t}}) & = & {{\rm{\psi }}}_{3}({\rm{t}}){{\rm{\varphi }}}_{3}({\rm{x}}),\end{array}$$

Where ϕ_1_ (ϕ_2_) is the localized state for the right (left) well of the lower dressed potential and ϕ_3_ is the localized state for the well of the upper dressed potential. ψ_j_(j = 1,2,3) is the corresponding time-dependent complex amplitude. Substituting the variational wave function (8) into the decoupled equations (7), one can get the nonlinear three-mode dynamical equations:

9 $$\begin{array}{rcl}{\rm{i}}\frac{{{\rm{d}}{\rm{\psi }}}_{1}}{{\rm{dt}}} & = & {{\rm{\varepsilon }}}_{1}{{\rm{\psi }}}_{1}-{{\rm{J}}{\rm{\psi }}}_{2}+\sum _{{{\rm{i}}}_{1}=1}^{2}\sum _{{{\rm{i}}}_{2}=1}^{2}\sum _{{{\rm{i}}}_{3}=1}^{2}{{\rm{U}}}_{1{{\rm{i}}}_{1}\leftrightarrow {{\rm{i}}}_{2}{{\rm{i}}}_{3}}{{\rm{\psi }}}_{{{\rm{i}}}_{1}}^{\ast }{{\rm{\psi }}}_{{{\rm{i}}}_{2}}{{\rm{\psi }}}_{{{\rm{i}}}_{3}}+\sum _{{\rm{j}}=1}^{2}{{\rm{U}}}_{13\leftrightarrow {\rm{j}}3}{|{{\rm{\psi }}}_{3}|}^{2}{{\rm{\psi }}}_{{\rm{j}}},\\ {\rm{i}}\frac{{{\rm{d}}{\rm{\psi }}}_{2}}{{\rm{dt}}} & = & {{\rm{\varepsilon }}}_{2}{{\rm{\psi }}}_{2}-{{\rm{J}}{\rm{\psi }}}_{1}+\sum _{{{\rm{i}}}_{1}=1}^{2}\sum _{{{\rm{i}}}_{2}=1}^{2}\sum _{{{\rm{i}}}_{3}=1}^{2}{{\rm{U}}}_{2{{\rm{i}}}_{1}\leftrightarrow {{\rm{i}}}_{2}{{\rm{i}}}_{3}}{{\rm{\psi }}}_{{{\rm{i}}}_{1}}^{\ast }{{\rm{\psi }}}_{{{\rm{i}}}_{2}}{{\rm{\psi }}}_{{{\rm{i}}}_{3}}+\sum _{{\rm{j}}=1}^{2}{{\rm{U}}}_{23\leftrightarrow {\rm{j}}3}{|{{\rm{\psi }}}_{3}|}^{2}{{\rm{\psi }}}_{{\rm{j}}},\\ {\rm{i}}\frac{{{\rm{d}}{\rm{\psi }}}_{3}}{{\rm{dt}}} & = & {{\rm{\varepsilon }}}_{3}{{\rm{\psi }}}_{3}+\sum _{{{\rm{i}}}_{1}=1}^{2}\sum _{{{\rm{i}}}_{2}=1}^{2}{{\rm{U}}}_{3{{\rm{i}}}_{1}\leftrightarrow {{\rm{i}}}_{2}3}{{\rm{\psi }}}_{{{\rm{i}}}_{1}}^{\ast }{{\rm{\psi }}}_{{{\rm{i}}}_{2}}{{\rm{\psi }}}_{3}+{{\rm{U}}}_{33\leftrightarrow 33}{|{{\rm{\psi }}}_{3}|}^{2}{{\rm{\psi }}}_{3},\end{array}$$

with the Josephson coupling strength

10 $${\rm{J}}=-\int {\rm{dx}}{{\rm{\varphi }}}_{1}^{\ast }[-\frac{1}{2}\frac{{\partial }^{2}}{\partial {{\rm{x}}}^{2}}+{{\rm{V}}}_{-}+{{\rm{V}}}_{{\rm{kin}}}]{{\rm{\varphi }}}_{2},\,$$

the zero-point energy for atoms in j-th mode

11 $${{\rm{\varepsilon }}}_{{\rm{j}}}=\{\begin{array}{c}\int {\rm{dx}}{{\rm{\varphi }}}_{{\rm{j}}}^{\ast }[-\frac{1}{2}\frac{{\partial }^{2}}{\partial {{\rm{x}}}^{2}}+{{\rm{V}}}_{{\rm{kin}}}+{{\rm{V}}}_{-}]{{\rm{\varphi }}}_{{\rm{j}}},(\,j=1,2),\\ \int {\rm{dx}}{{\rm{\varphi }}}_{{\rm{j}}}^{\ast }[-\frac{1}{2}\frac{{\partial }^{2}}{\partial {{\rm{x}}}^{2}}+{{\rm{V}}}_{{\rm{kin}}}+{{\rm{V}}}_{+}]{{\rm{\varphi }}}_{{\rm{j}}},(\,j=3),\end{array}$$

and the intra-mode and inter-mode exchange collision interaction strength

12 $${{\rm{U}}}_{{\rm{ij}}\leftrightarrow {\rm{kl}}}={\rm{g}}\int {\rm{dx}}{{\rm{\varphi }}}_{{\rm{i}}}^{\ast }{{\rm{\varphi }}}_{{\rm{j}}}^{\ast }{{\rm{\varphi }}}_{{\rm{k}}}{{\rm{\varphi }}}_{l},\,({\rm{i}},{\rm{j}},{\rm{k}},{\rm{l}}=1,2,3).$$

In the exchange collision denoted by ${\rm{ij}}\leftrightarrow {\rm{kl}}$, an atom from the k-th mode collides with an atom from the l-th mode and then these two atoms are transferred into an atom in the i-th mode and an atom in the j-th mode. For sufficiently deep wells, these three localized states are well localized at the corresponding well-centers, so that the high-order overlaps between the localized states are very small, namely

13 $${{\rm{U}}}_{{\rm{ij}}\leftrightarrow {\rm{kl}}}\ll {{\rm{U}}}_{{\rm{ii}}\leftrightarrow {\rm{ii}}},\,({\rm{i}},{\rm{j}},{\rm{k}},{\rm{l}}=1,2,3\,{\rm{and}}\,{\rm{at}}\,{\rm{least}}\,{\rm{one}}\,{\rm{of}}\,\{{\rm{j}},{\rm{k}},{\rm{l}}\}\,{\rm{not}}\,{\rm{equal}}\,{\rm{i}}).$$

Therefore the inter-mode exchange collision terms ${{\rm{U}}}_{{\rm{ij}}\leftrightarrow {\rm{kl}}}{{\rm{\psi }}}_{{\rm{j}}}^{\ast }{{\rm{\psi }}}_{{\rm{k}}}{{\rm{\psi }}}_{{\rm{l}}}$ in the three-mode dynamical equations (9) can be ignored, and Eq. (9) can be further simplified to be:

14 $$\begin{array}{rcl}{\rm{i}}\frac{{{\rm{d}}{\rm{\psi }}}_{1}}{{\rm{dt}}} & = & {{\rm{\varepsilon }}}_{1}{{\rm{\psi }}}_{1}-{{\rm{J}}{\rm{\psi }}}_{2}+{{\rm{U}}}_{11}{|{{\rm{\psi }}}_{1}|}^{2}{{\rm{\psi }}}_{1},\\ {\rm{i}}\frac{{{\rm{d}}{\rm{\psi }}}_{2}}{{\rm{dt}}} & = & {{\rm{\varepsilon }}}_{2}{{\rm{\psi }}}_{2}-{{\rm{J}}{\rm{\psi }}}_{1}+{{\rm{U}}}_{22}{|{{\rm{\psi }}}_{2}|}^{2}{{\rm{\psi }}}_{2},\\ {\rm{i}}\frac{{{\rm{d}}{\rm{\psi }}}_{3}}{{\rm{dt}}} & = & {{\rm{\varepsilon }}}_{3}{{\rm{\psi }}}_{3}+{{\rm{U}}}_{33}{|{{\rm{\psi }}}_{3}|}^{2}{{\rm{\psi }}}_{3},\end{array}$$

in which the intra-mode exchange collision interaction ${{\rm{U}}}_{{\rm{ii}}\leftrightarrow {\rm{ii}}}\,({\rm{i}}=1,2,3)$ has been expressed as U_ii_ for simplicity.

In the following, we will consider both the Bose-Josephson (BJ) dynamics and LZ dynamics and study the impact of the coupling between dressed states on the dressed state dynamics in our system.

### Bose-Josephson dynamics

BJ effect is a peculiar tunneling phenomenon in the physical system of two BECs linked by Josephson coupling^30–33^. In our coupled two-component BEC system, when the inter-component coupling has modest strength, the lower dressed potential has a double-well structure. In analogy with the conventional BJ junctions, BJ dynamics will occur in the dressed state picture. When considering a weak interacting BEC (g < g_*c*_), the Josephson oscillations in the lower dressed state are observed. When taking into account a BEC with stronger atom-atom interaction (g > g_*c*_), the MQST in the lower dressed state will take place. Here, g_*c*_ is the critical interaction strength between the normal phase (i.e. the Josephson oscillations) and the MQST phase of the lower dressed state. Remarkably, different from BJ dynamics in an external BJ junction or an internal BJ junction^30,33^, in our system the BEC bulk oscillates not only between two external modes but also two internal states. In the following, we will discuss the Josephson oscillations and MQST dynamics for the dressed states and determine the critical value g_*c*_ by both analytic and numerical methods.

**Figure 2:**
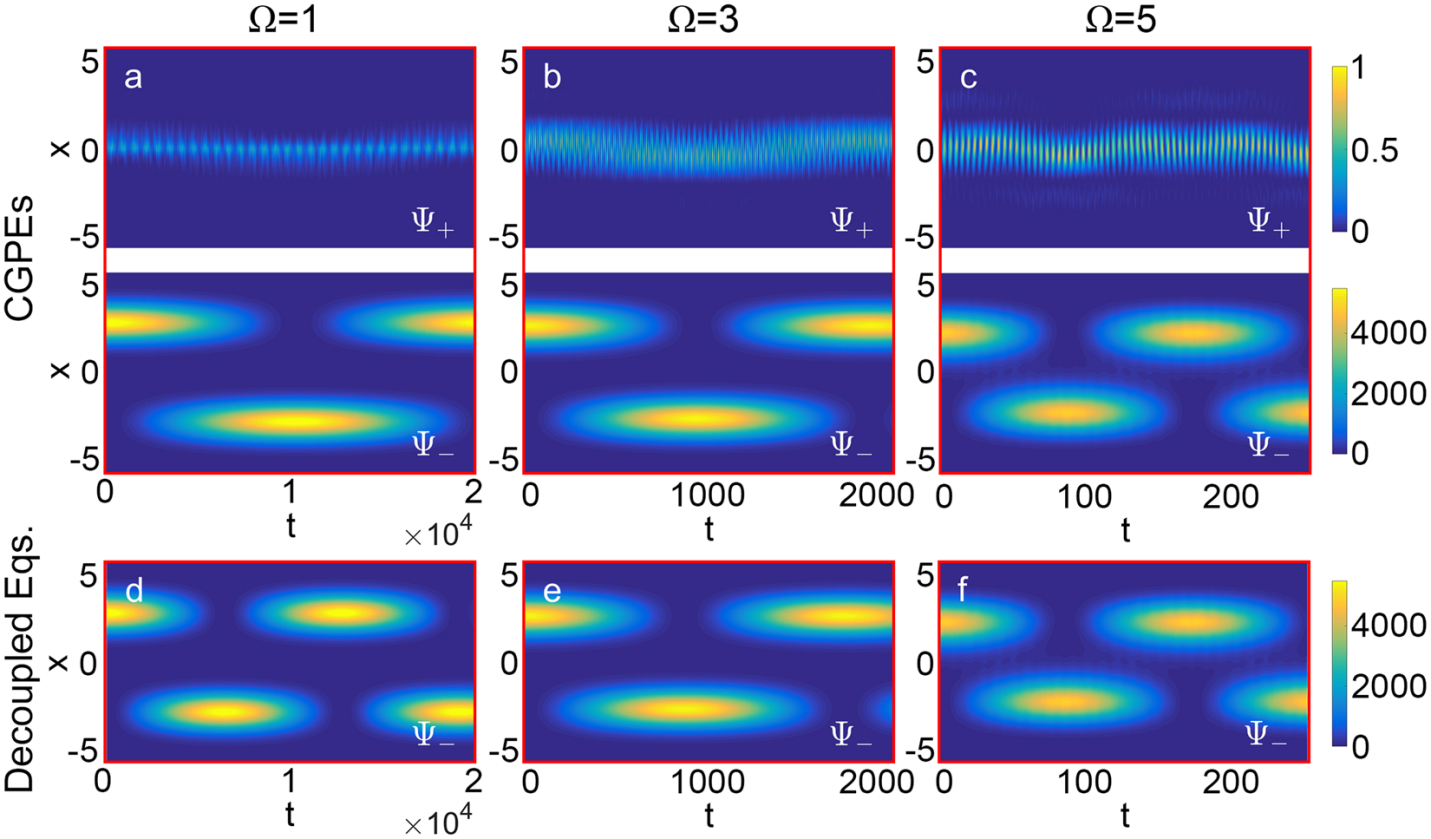
The Bose-Josephson dynamics of the dressed states for non-interacting BEC. (**a**–**c**) The simulation results of Josephson oscillations via the CGPEs [Eq. (5)] for different coupling strength Ω. The figures in the first (second) row are the time evolution of the density profiles for upper (lower) dressed state, denoted by ${{\rm{\Psi }}}_{+}$ (${{\rm{\Psi }}}_{-}$). (**d**–**f**) The simulation results of Josephson oscillations for lower dressed state Ψ _−_, calculated by the decoupled equations [Eq. (7)] for different coupling strength Ω. The initial state is set as the located state of the right well of the lower dressed potential, namely Ψ_−_(x, t = 0) = ϕ_1_(x) and Ψ_+_(x, t = 0) = 0, see Fig. 1(b) (I). Other parameters: atom number N = 10000, interaction strength g = 0, potential separation x_0_ = 3.

At first, for non-interacting BEC (g = 0), we simulate the dressed state dynamics with different coupling strength Ω by solving the CGPEs (5). We set the initial state as the located state of the right well of the lower dressed potential:

15 $${{\rm{\Psi }}}_{-}({\rm{x}},{\rm{t}}=0)={{\rm{\varphi }}}_{1}({\rm{x}}),{{\rm{\Psi }}}_{+}({\rm{x}},{\rm{t}}=0)=0,$$

see Fig. 1(b) (I). The wave packet of the lower dressed state will tunnel from the right well to the left well and vice versa due to the BJ effect. With fixed x_0_, the barrier between two wells become lower as Ω increases, and the period of the Josephson oscillations will decrease quickly, from T ≈ 21000 (for Ω = 1) to T ≈ 180 (for Ω = 5), see Fig. 2(a–c). The average population imbalance between the two wells over a long time-evolution tends to be zero.

When the gap between the upper and lower dressed potentials is large enough, the coupling between the upper and lower dressed states will be so weak that can be neglected. Therefore, the BJ dynamics for the lower dressed state can be captured by the decoupled equations (7). In Fig. 2(d**–**f), we provide the simulation results based on the decoupled equations (7) for Ω = 1, 3, 5. Comparing with the numerical results based on the CGPEs (5) [Fig. 2(a–c)], the decoupled equations (7) are in accordance when Ω is large (e.g., Ω = 3, 5). While for small Ω (e.g., Ω = 1), the time-evolution of the density profile for lower dressed state obtained by the decoupled equations (7) is quite different from the original one calculated by the CGPEs (5). The main difference reflects on the period of the Josephson oscillations T. The decoupled equations (7) predict smaller period when Ω is small.

**Figure 3:**
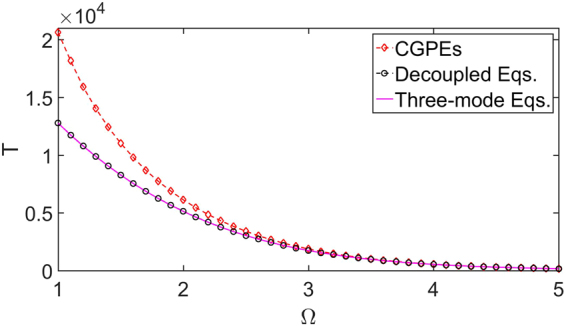
The periods of Josephson oscillations for non-interacting BEC obtained via different methods. The red diamonds and black circles indicate the Josephson oscillations periods versus coupling strength Ω, obtained via the CGPEs [Eq. (5)] and decouple equations [Eqs. (7)] respectively. The purple line is obtained from the solution of the three-mode equation (14) in the linear regime, i.e. T = π/J. Other parameters: interaction strength g = 0, spatial separation x_0_ = 3.

To investigate the validity of the decoupling approximation, we compare the period of the Josephson oscillations obtained from the CGPEs (5) and the decoupled equations (7) under different Ω, see Fig. 3. It is shown that, the results of the decoupled equations (7) accord well with the original one of the CGPEs (5) when Ω is large. As Ω decreases, the results of the decoupled equations (7) gradually deviate from the original one, which is due to the increasing coupling between the two dressed states. Specifically, we assume that if the difference between the periods obtained from the CGPEs (5) and those obtained from the decoupled equations (7) is below a threshold 5%, the decoupling approximation is judged to be valid. According to the criteria, we find the decoupling approximation is valid when 3.3 ≤ Ω ≤ 5 for fixed x_0_ = 3.

In addition, the period of Josephson oscillation T can also be obtained by solving the three-mode equations (14) in linear regime, namely T = π/J. In Fig. 3, we also plot the results and it agrees perfectly with the results of the decoupled equations (7) when 0 ≤ Ω ≤ 5.

Interestingly, when we consider the effects of atom-atom interaction, the nonlinearity will gradually affect the Josephson oscillations. Still, we locate the initial wave packet at the center of the right well of the lower dressed potential:

16 $${{\rm{\Psi }}}_{-}({\rm{x}},{\rm{t}}=0)={\varphi }_{1}({\rm{x}}),{{\rm{\Psi }}}_{+}({\rm{x}},{\rm{t}}=0)=0.$$

**Figure 4:**
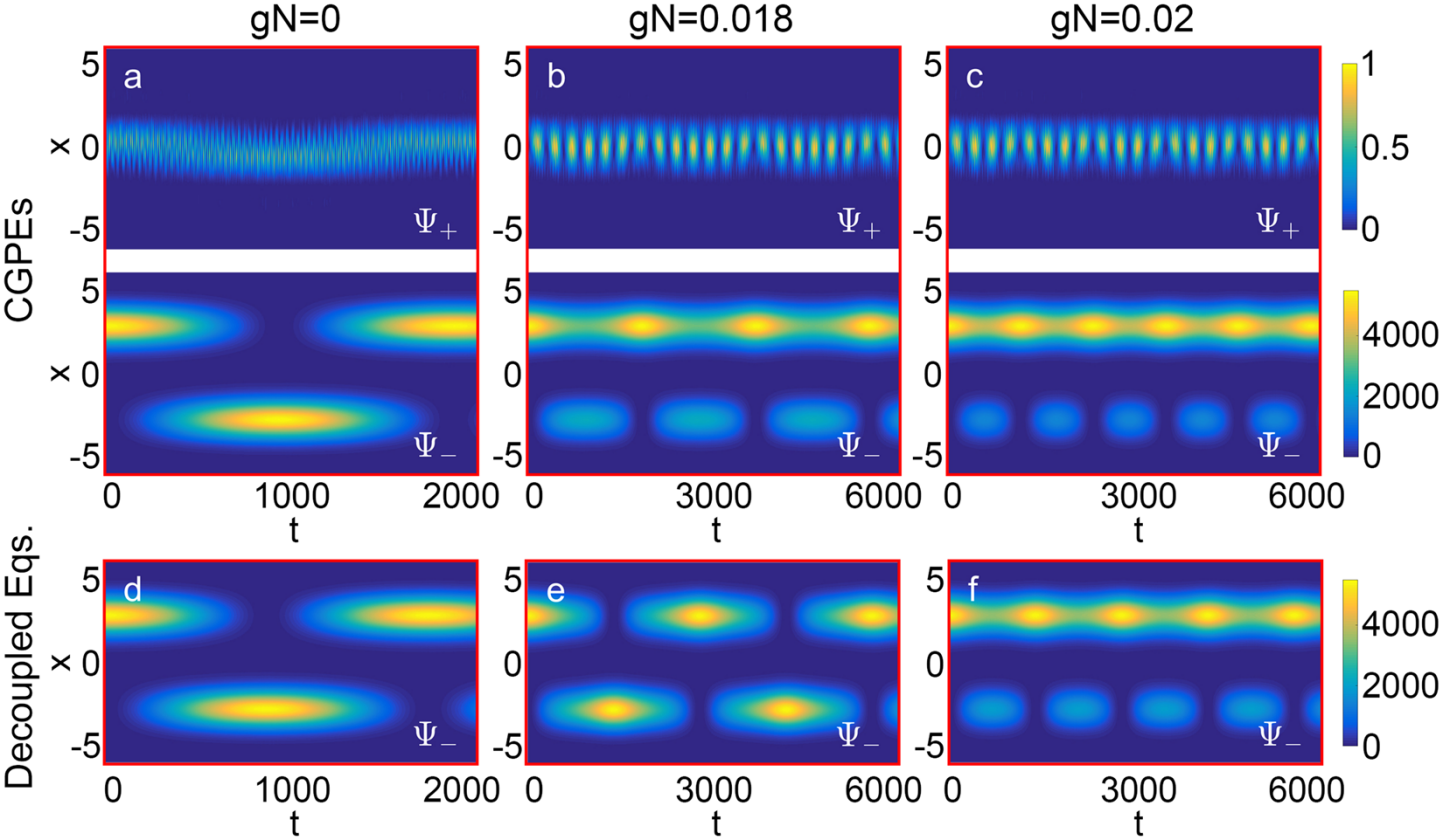
The normal-to-MQST phase transition of dressed states for interacting BEC. (**a**–**c**) The simulation results via the CGPEs [Eq. (5)]. (**a**) Josephson oscillations. (**b**), (**c**) MQST appears as the interaction strength increases. The initial state is set as the localized state of the right well of the lower dressed potential, namely Ψ_−_(x, t = 0) = ϕ_1_(x) and Ψ_+_(x, t = 0) = 0, see Fig. 1(b) (**I**). The figures in the first (second) row denote the time evolution of the density profiles for upper (lower) dressed state, labeled by ${{\rm{\Psi }}}_{+}$(${{\rm{\Psi }}}_{-}$). The normal-to-MQST transition happens when interaction strength is approximately gN = 0.018. (**d**–**f**) The simulation results obtained by the decoupled equations [Eq. (7)]. For this method, the normal-to-MQST transition happens when interaction strength is between gN = 0.018 and gN = 0.02. Other parameters: atom number N = 10000, potential separation x_0_ = 3, and coupling strength Ω = 3.

To see the impact of atom-atom interaction, as an example, we plot the time evolution of density profiles for the dressed states with x_0_ = 3 and Ω = 3 under gN = 0, 0.018 and 0.02, see Fig. 4(a–c). For gN = 0.018, MQST occurs in the lower dressed state with the average population imbalance between the two wells becoming nonzero. The lower dressed state prefers to stay in the initially populated well as the interaction plays a role. When gN increases, MQST begins to dominate and the oscillations between the two wells are gradually suppressed.

The coupling between the dressed states will influence the critical condition from Josephson oscillations to MQST. Here, we also use the decoupled equations (7) to simulate the BJ dynamics for the lower dressed state in the nonlinear regime. The results of decoupled equations (7) are slightly different from the original ones. For example, when gN = 0.018, MQST appears in the simulation results of the CGPEs (5) while the corresponding results of the decoupled equations (7) still show Josephson oscillations. For decoupled equations (7), the MQST occurs when gN ≈ 0.02, which is larger than the original critical point.

**Figure 5:**
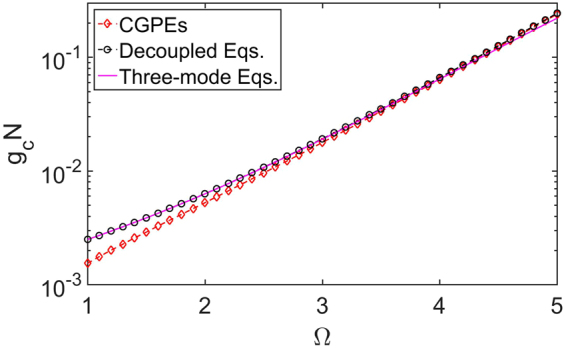
The critical interaction strength of normal-to-MQST transition for dressed states. The red diamonds and black circles indicate the critical interaction strength of phase transition versus the coupling strength Ω, which are obtained from the CGPEs [Eq. (5)] and the decoupled equations [Eq. (7)], respectively. The purple line is the analytical result [Eq. (18)] calculated from the three-mode equation [Eq. (14)]. Other parameters: atom number N = 10000, spatial separation x_0_ = 3.

The critical point of gN for the transition from Josephson oscillations to MQST obtained by the CGPEs (5) and the decoupled equations (7) for different Ω are shown in Fig. 5. Also, we assume that if the difference between the results obtained from the CGPEs (5) and those obtained from the decoupled equations (7) is below a threshold 5%, the decoupling approximation is regarded as valid. According to the criteria, it is shown that the decoupled equations (7) can give a relatively correct critical interaction strength when 3.4 ≤ Ω ≤ 5, see Fig. 5. However, when Ω becomes small enough, the results deviate from the results of the CGPEs (5).

The MQST can also be explained by the solution of the stationary states of the three-mode equations (14). The complex amplitudes in the three-mode equations (14) can be expressed as ${{\rm{\psi }}}_{{\rm{j}}}({\rm{t}})=\sqrt{{{\rm{n}}}_{{\rm{j}}}}\exp ({{\rm{i}}{\rm{\theta }}}_{{\rm{j}}})$, where n_j_ is the atom number and θ_j_ is the phase. For the two dressed down modes, we introduce two new variables, the relative population imbalance z = (n_1_ − n_2_)/N and the phase difference θ = θ_1_ − θ_2_. By inserting them into (14), (θ, z) obey:

17 $$\begin{array}{rcl}\frac{d{\rm{\theta }}}{{\rm{dt}}} & = & \frac{{{\rm{E}}}_{{\rm{c}}}{\rm{Nz}}}{2}+\frac{2{\rm{Jz}}}{\sqrt{1-{{\rm{z}}}^{2}}}\,\cos \,{\rm{\theta }},\\ \frac{{\rm{dz}}}{{\rm{dt}}} & = & -2{\rm{J}}\sqrt{1-{{\rm{z}}}^{2}}\,\sin \,{\rm{\theta }},\end{array}$$

in which $${{\rm{E}}}_{{\rm{c}}}={{\rm{U}}}_{11}+{{\rm{U}}}_{22}$$.

Mathematically, fixed points for (θ, z) are determined by $\frac{d{\rm{\theta }}}{{\rm{dt}}}=0$ and $\frac{{\rm{dz}}}{{\rm{dt}}}=0$. In the linear regime, namely E_c_ = 0, there are two normal fixed points (θ^*^ = 0, z^*^ = 0) and (θ^*^ = π, z^*^ = 0), and the general solutions are sinusoidal oscillations around one of these fixed points with frequencies ω_R_ = 2J. In the nonlinear regime, i.e., E_c_ ≠ 0, as the nonlinear interaction strength increases and exceeds a threshold

18 $${{\rm{E}}}_{{\rm{c}}}^{2}{{\rm{N}}}^{2} > 16{{\rm{J}}}^{2},$$

there exist two new fixed points: $({{\rm{\theta }}}^{\ast }={\rm{\pi }},{{\rm{z}}}^{\ast }=\pm \sqrt{1-\frac{16{{\rm{J}}}^{2}}{{{\rm{E}}}_{{\rm{c}}}^{2}{{\rm{N}}}^{2}}})$ when E_c_ < 0; $({{\rm{\theta }}}^{\ast }=0,{{\rm{z}}}^{\ast }=\pm \sqrt{1-\frac{16{{\rm{J}}}^{2}}{{{\rm{E}}}_{{\rm{c}}}^{2}{{\rm{N}}}^{2}}})$ when E_c_ < 0. The corresponding general solutions are oscillations enclosing these fixed points. Different from the general solutions in the linear regime, they have nonzero average population imbalances, which indicate the MQST. In Fig. 5, it is shown that the critical interaction strength of normal-to-MQST transition calculated analytically with Eq. (18) is well consistent with the numerical results of decoupled equations (7) when Ω is not too large. However, when Ω is large enough, the wells in the lower dressed potential are too shallow to ensure the accuracy of the three-mode approximation. We assume that if the difference between the critical interaction strength obtained by the decoupled equations (7) and the analytical results (18) is below a threshold 5%, the three-mode approximation is regarded as valid. For fixed potential separation x_0_ = 3, we find the reasonable range of the three-mode approximation is 0 ≤ Ω ≤ 4.5, see Fig. 5.

It is obvious that, the decoupled equations (7) are valid when the coupling strength is large enough. Based on our analytic and numerical results, for both non-interacting and interacting BEC, only when the gap between the upper and lower dressed potentials is sufficiently large, the lower dressed double-well potential can be treated as adiabatic potential and the BJ dynamics can be characterized by the decoupled equations (7). In addition, if the wells in the dressed potential is not too shallow (i.e. Ω is not too large), the three-mode approximation is suitable to describe the system.

### Landau-Zener dynamics

In our coupled two-component BEC system, even when the coupling strength Ω is large enough, the atoms may still transfer from an adiabatic potential to the other so that the adiabaticity is not guaranteed. In general, this can happen when an atom accumulates large enough kinetic energy so that it cannot follow the adiabatic potential. Therefore, the dynamics of the dressed states depend not only on the structure of the dressed potentials but also on the initial state. Since the boundaries of the potentials can be sufficiently high, the initial wave packet that far away from the minimum of the lower dressed potential would acquire a large kinetic energy. The kinetic energy enables the transition between the lower and upper dressed states at the crossing point x = 0, which is the LZ transitions. It is necessary to determine the conditions in which the LZ transition is negligible since the technique of adiabatic potential is always used in the limits of adiabaticity.

To observe the LZ dynamics for the dressed states, one can set the initial state as a wave packet in the lower dressed state centering at a position away from the minimum of the lower dressed potential with a distance x_d_:

19 $${{\rm{\Psi }}}_{-}({\rm{x}},{\rm{t}}=0)={\varphi }_{1}({\rm{x}}-{{\rm{x}}}_{{\rm{d}}}),\,{{\rm{\Psi }}}_{+}({\rm{x}},{\rm{t}}=0)=0$$

**Figure 6:**
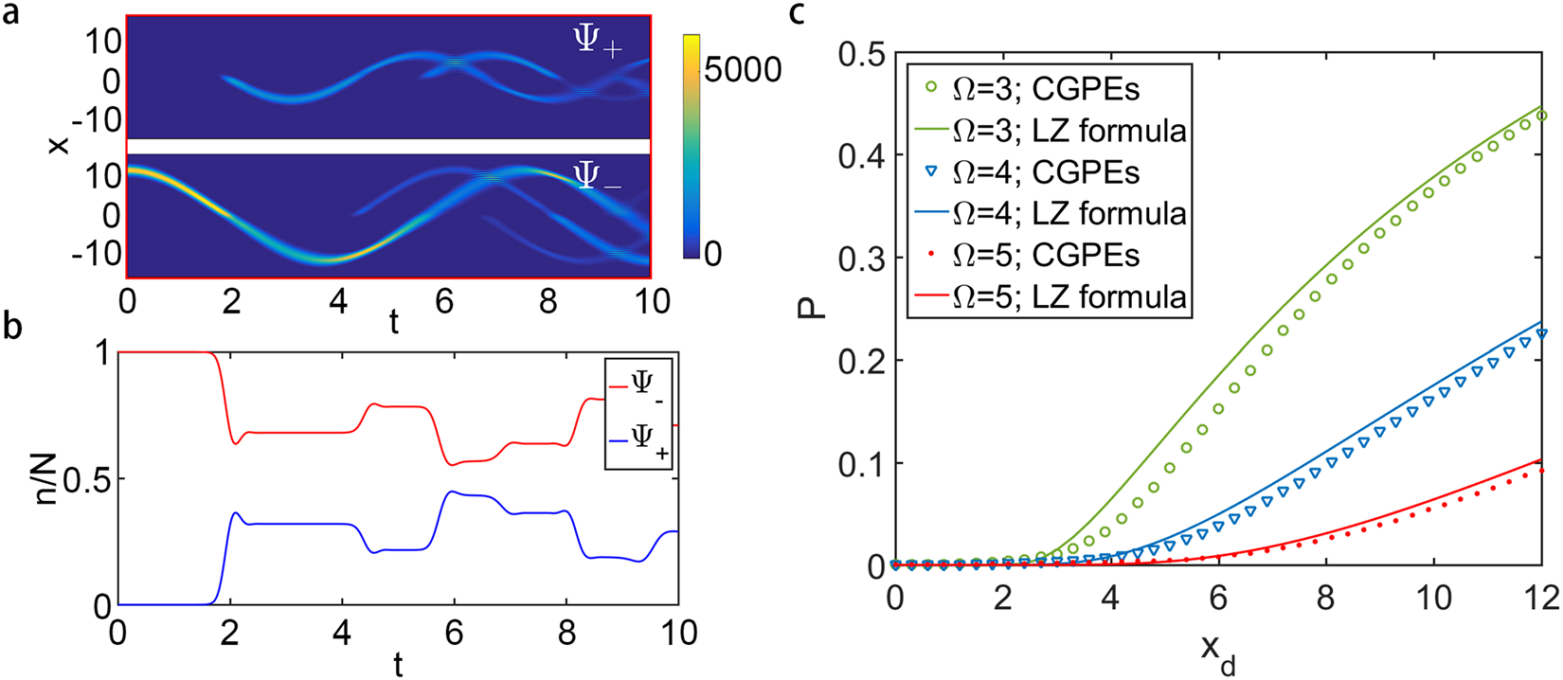
Landau-Zener dynamics. (**a**,**b**) Time evolution of the density profile and the population for the upper (lower) dressed state, labeled by ${{\rm{\Psi }}}_{+}$ (${{\rm{\Psi }}}_{-}$), obtained by solving the CGPEs [Eq. (5)]. The initial state is set as a wave packet in lower dressed state centering at a position away from the minimum of the right well of the lower dressed potential with a distance x_d_, namely, Ψ_−_(x, t = 0) = *ϕ*_1_(x − x_d_) and Ψ_+_(x, t = 0) = 0; see Fig. 1(b) (II). The coupling strength is Ω = 3. (**c**) Non-adiabatic transition probability P obtained from the original CGPEs [Eq. (5)] (dots) and the LZ formula [Eq. (23)] (solid lines) for different coupling strength Ω and x_d_. Other parameters: interaction strength g = 0, potential separation x_0_ = 3.

See Fig. 1(b) (II). The time evolution of the density profile for the dressed states given by the CGPEs (5) is shown in Fig. 6(a). One can find that the dynamics are dominated by the harmonic oscillations and the LZ transitions of the dressed states. During the evolution, when the bulk of BECs reaches the avoided-crossing at x = 0, the LZ transitions occur and part of the atoms in the lower (upper) dressed state are transferred to the upper (lower) dressed state, see Fig. 6(b).

The non-adiabatic transition probability of the LZ transition depends on the two-component coupling strength Ω and the wave packet’s initial position. The non-adiabatic transition probability P can be obtained numerically from the simulation results of the CGPEs (5). The results of non-adiabatic transition probability P for different coupling strength Ω and initial wave packet position are shown in Fig. 6(c). One can find that the non-adiabatic transition probability P decreases as the coupling strength Ω increases, which is due to the decreasing of the coupling coefficients C_1_ and C_2_. On the other hand, if the initial wave packet approaches the potential minima, which corresponds to a lower potential energy, the wave packet will get a smaller velocity near the avoided-crossing and the terms ${\partial }_{{\rm{x}}}{{\rm{\Psi }}}_{-}$, ${\partial }_{{\rm{x}}}{{\rm{\Psi }}}_{+}$ in the dressed state coupling terms (6) will decrease, resulting in the decrease of P.

One can also derive the non-adiabatic transition probability P by the LZ formula. The transition only occurs in the small region near the avoided-crossing, so that we may treat the velocity of the wave packet v as constant during the LZ transition. The initial wave packet can be decomposed to different momentum components by Fourier transforms:

20 $${{\rm{\Phi }}}_{-}({\rm{k}})=\frac{1}{\sqrt{2{\rm{\pi }}}}\int {\rm{dx}}{\varphi }_{1}({\rm{x}}-{{\rm{x}}}_{{\rm{d}}}){{\rm{e}}}^{-{\rm{ikx}}}.$$

By treating the BEC atoms classically, the magnitude of the velocity v at the avoided-crossing for each momentum components can be determined by:

21 $$\frac{1}{2}{{\rm{v}}}^{2}+{{\rm{V}}}_{-}(0)=\frac{1}{2}{{\rm{k}}}^{2}+{{\rm{V}}}_{-}({{\rm{x}}}_{{\rm{m}}}+{{\rm{x}}}_{{\rm{d}}}),$$

in which x_m_ is the position of the right minima of the lower dressed potential. According to the LZ formula^34^, the non-adiabatic transition probability for each momentum components is

22 $$\begin{array}{rcl}{{\rm{P}}}_{{\rm{LZ}}}({\rm{k}}) & = & {{\rm{e}}}^{-2{\rm{\pi }}{\rm{\Gamma }}({\rm{k}})},\\ {\rm{\Gamma }}({\rm{k}}) & = & \frac{{{\rm{\Omega }}}^{2}}{|{\rm{v}}({\rm{k}}){[\frac{\partial }{\partial {\rm{x}}}({{\rm{V}}}_{1}-{{\rm{V}}}_{2})]|}_{{\rm{x}}=0}|}.\end{array}$$

Therefore, the total non-adiabatic transition probability can be obtained by integrating the momentum space:

23 $${\rm{P}}=\frac{1}{{\rm{N}}}{\int }^{}{\rm{dk}}{|{{\rm{\Phi }}}_{-}({\rm{k}})|}^{2}{{\rm{P}}}_{{\rm{LZ}}}({\rm{k}}).$$

The non-adiabatic transition probabilities calculated from the LZ formula with different initial location are shown by solid lines in Fig. 6(c), which agree well with the numerical results obtained from the CGPEs (5) [shown by the markers in Fig. 6 (c)].

Therefore, to guarantee the adiabaticity of the dressed potential and study solely the dynamics of lower dressed state, not only the coupling strength Ω should be large enough, but also the initial wave packet should be located near the minima of the double-well. The valid range of adiabaticity can be obtained from both the numeric results given by the CGPEs (5) and the analytical results given by the LZ formula (23). Specifically, we assume that if the non-adiabatic transition probability P is below a threshold 1%, the LZ transition can be neglected during the dynamics. Therefore, to avoid the LZ transition in practice, the distance x_d_ between the center of initial wave packet and the potential minima should be less than 3.0, 4.5, 6.3 when Ω = 3, 4 and 5 respectively, see Fig. 6(c). That is, as the coupling strength increases, the LZ transition is suppressed and the valid distance x_d_ increases. These results give an instruction for experimental realization of adiabatic potentials to avoid the influences of LZ transitions.

## Summary and Discussion

In summary, we have investigated BJ dynamics and LZ dynamics for the dressed states in coupled two-component BECs in a state-dependent potential. In our system, a double-well structure can be formed in the lower dressed potential. The dressed state dynamics sensitively depend on both the inter-component coupling strength and the choice of initial state. We discuss how the BJ dynamics and LZ dynamics are affected by the inter-component coupling strength and the initial state.

If the initial state is prepared at the minimum of the lower dressed potential, one can observe BJ dynamics in the dressed state. When the inter-component coupling strength is large enough, the dressed states can be treated as decoupled. As the nonlinear interaction comes into play, Josephson oscillations would gradually transit to MQST, and the critical condition for the transition can be well described by the three-mode approximation.

If the initial state is far away from the minimum of the lower dressed potential, there will appear several LZ transitions at the avoided-crossing point even though the gap is large. The LZ transitions between the dressed states well obey the LZ formula. To ensure the adiabaticity of the dressed potential, not only the inter-component coupling strength should be strong enough but also the initial wave packet should not locate too far away from the potential minima.
